# Uric Acid Protects against Focal Cerebral Ischemia/Reperfusion-Induced Oxidative Stress via Activating Nrf2 and Regulating Neurotrophic Factor Expression

**DOI:** 10.1155/2018/6069150

**Published:** 2018-11-18

**Authors:** Bai-liu Ya, Qian Liu, Hong-fang Li, Hong-ju Cheng, Ting Yu, Lin Chen, You Wang, Li-li Yuan, Wen-juan Li, Wen-yan Liu, Bo Bai

**Affiliations:** ^1^Department of Physiology, Jining Medical University, Shandong 272067, China; ^2^School of Clinical Medicine, Jining Medical University, Shandong 272067, China; ^3^Department of Neurology, Affiliated Hospital of Jining Medical University, Shandong 272067, China; ^4^School of Basic Medicine, Jining Medical University, Shandong 272067, China; ^5^School of Forensic and Laboratory Medicine, Jining Medical University, Shandong 272067, China

## Abstract

The aim of this study was to investigate whether uric acid (UA) might exert neuroprotection via activating the nuclear factor erythroid 2-related factor 2 (Nrf2) pathway and regulating neurotrophic factors in the cerebral cortices after transient focal cerebral ischemia/reperfusion (FCI/R) in rats. UA was intravenously injected through the tail vein (16 mg/kg) 30 min after the onset of reperfusion in rats subjected to middle cerebral artery occlusion for 2 h. Neurological deficit score was performed to analyze neurological function at 24 h after reperfusion. Terminal deoxynucleotidyl transferase-mediated dNTP nick end labeling (TUNEL) staining and hematoxylin and eosin (HE) staining were used to detect histological injury of the cerebral cortex. Malondialdehyde (MDA), the carbonyl groups, and 8-hydroxyl-2′-deoxyguanosine (8-OHdG) levels were employed to evaluate oxidative stress. Nrf2 and its downstream antioxidant protein, heme oxygenase- (HO-) 1,were detected by western blot. Nrf2 DNA-binding activity was observed using an ELISA-based measurement. Expressions of BDNF and NGF were analyzed by immunohistochemistry. Our results showed that UA treatment significantly suppressed FCI/R-induced oxidative stress, accompanied by attenuating neuronal damage, which subsequently decreased the infarct volume and neurological deficit. Further, the treatment of UA activated Nrf2 signaling pathway and upregulated BDNF and NGF expression levels. Interestingly, the aforementioned effects of UA were markedly inhibited by administration of brusatol, an inhibitor of Nrf2. Taken together, the antioxidant and neuroprotective effects afforded by UA treatment involved the modulation of Nrf2-mediated oxidative stress and regulation of BDNF and NGF expression levels. Thus, UA treatment could be of interest to prevent FCI/R injury.

## 1. Introduction

The principal therapy for cerebral ischemia is the restoration of cerebral blood flow as early as possible. Early recanalization with recombinant tissue plasminogen activator (rt-PA) has been developed to treat acute ischemic stroke. However, reperfusion after cerebral ischemia may be injurious and increase the risk of brain hemorrhage, promote the development of cerebral edema, and cause more serious damage to the blood-brain barrier. The potential mechanisms responsible for the additional injuries in the ischemic brain caused by reperfusion itself remain unclear [1]. Acute cerebral ischemia starts with cerebral blood flow interruption that causes severely limited oxygen and glucose supply, eliciting a cascade of pathological events such as excitotoxicity, calcium dysregulation, oxidative stress, and inflammatory that could ultimately result in tissue death. Oxidative stress is linked with a progressive increase in reactive oxygen species (ROS), and it affects the pathogenesis of cerebral ischemia/reperfusion (I/R) injury seriously [2–4]. Oxidative insult after I/R injury increases pathological alteration of lipids, proteins, and DNA, thereby damaging function of the cell, which lastly causes the cell death. Enhanced ROS production after reperfusion increases hemorrhagic infarction, brain edema, and infarction size. So the intervention of oxidative damage using safe and effective therapeutic agents with antioxidant properties provides an encouraging therapeutic strategy.

Phase 2 enzymes have been implicated to be the important means by which neurons protect themselves against intense oxidative stress. The expression of phase 2 enzymes, including heme oxygenase (HO)-1, is regulated by nuclear factor E2-related factor 2 (Nrf2) [5]. Considerable efforts have been made to develop effective strategies and drugs to relieve or prevent cerebral I/R injury. Extensive research has confirmed that Nrf2 activation during I/R is becoming a promising therapeutic target for neuroprotection [6, 7]. We also have recently indicated a key role of Nrf2 activation in prevention of ischemic cerebrovascular disease in our previous studies [8].

Neurotrophic factors are essential regulators in poststroke recovery [9–11]. Both brain-derived neurotrophic factor (BDNF) and nerve growth factor (NGF) have been reported to be neuroprotective in the middle cerebral artery occlusion (MCAO) rat model of ischemia [12, 13]. Previous experimental studies have demonstrated that these neurotrophic factors confer neuroprotective effects as important candidates for prevention of oxidative stress and subsequent neurotoxicity [14, 15]. The expression levels of neurotrophic factors such as BDNF and NGF, which are causally related to oxidative stress, exert key effects in keeping the balance between oxidation and antioxidation mechanisms. Both BDNF and NGF are Nrf2-target genes [16]. In addition, it has been demonstrated that neurotrophins can activate Nrf2 [17–19]. So our choice of regulator molecules, BDNF and NGF, and the transcription factor, Nrf2, which participate in multiple steps of the active process of oxidative stress, is justified because dysfunction of any of these proteins causes a redox imbalance that leads to oxidative damage.

Uric acid (UA) is a major antioxidant in the blood in humans and is responsible for almost two-thirds of all free radical scavenging capacity with a concentration that surpasses other antioxidants by as much as tenfold in plasma [20]. Since UA has a wide variety of profound antioxidant properties, including quenching superoxide and hydroxyl and peroxynitrite radicals, and may have beneficial effects via reducing lipid peroxidation [21, 22]; it should have protective effects against ischemic brain damage. Actually, there are several animal experimental data in which the neuroprotective property of exogenous administration of uric acid is demonstrated [23–27]. Evidence has been presented that UA can exert much of its neuroprotective effect by astrocytic Nrf2/antioxidant response element (ARE) pathway activation indirectly causing increased levels of glutathione concentration [28], which implies that Nrf2 pathway may participate in the neuroprotection of UA. However, it remains unknown whether the Nrf2 pathway activation is involved in the UA-induced neuroprotection against the cerebral I/R injury via neuronal antioxidation approach and whether neurotrophic factors are involved in the UA's neuroprotection against oxidative damage after stroke. Therefore, we employed the MCAO-induced focal cerebral I/R (FCI/R) rat model to test the hypothesis that UA protects against FCI/R-induced oxidative stress injury by activating Nrf2 pathway and targeting BDNF and NGF expression. Furthermore, this study may help understanding the relationship between Nrf2 signaling activation and the roles of BDNF and NGF in the pathogenesis of the ischemic brain in the neuroprotection induced by UA.

## 2. Materials and Methods

### 2.1. FCI/R Rat Model

Male Sprague–Dawley (SD) rats (280–300 g) from Pengyue Experimental Animal Breeding Institute of Jinan, China, were maintained in a room (23-24°C, 50-60% humidity) under 12 h light–dark cycle with free access to water and food. All experimental protocols were conducted according to ARRIVE (Animal Research: Reporting In Vivo Experiments) guidelines. Every effort was made to minimize animal suffering and the number of animals used. The Ethics Committee of Jining Medical University approved all the experimental protocols. All sample analysis was conducted under a strictly blinded manner. 193 rats were used, of which 11 died after transient FCI/R insult and 2 died after left lateral ventricle puncture, and consequently a total of 180 rats were separated randomly into 4 groups which included the sham group (*n* = 45), vehicle-treated I/R group (*n* = 45), 16 mg/kg UA-treated I/R group (UA group, *n* = 45), and UA plus brusatol-treated I/R group (UA + Bru, a specific inhibitor of Nrf2, *n* = 45).

The procedure for transient FCI/R has been described previously [29]. For MCAO, a 4-0 nylon thread with a rounded tip was inserted into the left external carotid artery (CA) and subsequently went into the internal CA approximately 20–21 mm from the carotid bifurcation. Then, the thread was withdrawn to initiate reperfusion after 2 h ischemia. The left CA was isolated with no thread inserted in the sham group. Rectal temperature was monitored and maintained between 37.0 and 37.5°C with a heating pad throughout surgery.

### 2.2. Drug Treatment and Experimental Design

UA diluted with Locke's buffer (vehicle) was injected intravenously through the tail vein (16 mg/kg) 30 min after reperfusion. An equal volume of vehicle was given to sham and model rats. Rats in the UA + Bru group were treated with UA plus brusatol. The dose of UA was chosen based on previous reports of UA treatment in rats induced by transient FCI/R [24–26]. Left lateral ventricle puncture (anteroposterior, −0.9 mm; lateral, 1.5 mm; depth, 3.6 mm; from bregma) was performed before MCAO. In the UA + Bru group, rats were infused intracerebroventricularly with Nrf2 inhibitor brusatol 30 min prior to ischemia (1 mg/kg, dissolved in 5 *μ*l 1% DMSO) [30]. The other three groups also received equal volume vehicle injection after surgery. After animals underwent neurological evaluation at 24 h of reperfusion, their brains were harvested for the experiments described below.

### 2.3. Measurement of Cerebral Infarct Area

2,3,5-Triphenyltetrazolium chloride (TTC) staining technique was employed to test the cerebral infarct volume. TTC stains the noninfarcted region with a deep red pigment, while the infarcted brain area appears white. Infarct volume was calculated with correction of edema in the ischemic hemisphere as previously described [29].

### 2.4. Evaluation of Neurological Deficits

A blinded observer evaluated the neurological status 24 h after FCI/R. A 5-point scale was used as follows [31, 32]: 0, no neurological deficits; 1, failure to fully extend the right forepaw; 2, decreased resistance to a lateral push toward the left side and failure to fully extend the right forepaw; 3, decreased resistance to a lateral push toward the left side, failure to fully extend the right forepaw, and circling to the left side; and 4, inability to walk spontaneously and lack of response to stimulation.

### 2.5. Determination of Brain Edema

The brains were carefully removed after decapitation. The wet weight was obtained immediately by weighing the ischemic hemispheres. The dry weight was obtained by weighing the samples dried at 60°C for 72 h. The percentage tissue water content was determined as previously described [8].

### 2.6. Histology and Immunochemistry Analysis

Rats were transcardially perfused with PBS and 4% paraformaldehyde 24 h after transient FCI/R (*n* = 3/group). The brain coronal sections from bregma at 0.3 to −1.8 mm were cut at 20 *μ*m thickness. The sections were stained for hematoxylin and eosin (HE), terminal deoxynucleotidyl transferase- (TdT-) mediated dNTP nick end labeling (TUNEL), the expression of BDNF and NGF, and 8-hydroxy-deoxyguanosine (8-OHdG). Sections were incubated with primary antibody (anti-BDNF, Santa Cruz, USA; anti-NGF, Abcam Inc., Cambridge, MA; anti-8-OHdG, JaICa, Shizuoka, Japan) and then with the biotinylated secondary antibody. Negative controls performed the same staining procedure with the omission of primary antibody. HE staining was performed according to the standard procedure. TUNEL labeling assay kit (Roche Company, Switzerland) was used to measure DNA fragmentation. The number of HE-, TUNEL-, BDNF-, NGF-, and 8-OHdG-positive cells in the penumbra of ischemic ipsilateral parietal cortex in the ischemic area was quantified under a 400x magnification by a blinded investigator.

### 2.7. Western Blot

The ischemic side cerebral cortex (left side, *n* = 4/group) was dissected to extract total protein using a total protein extraction kit (Applygen Technologies Inc., Beijing). A nuclear-cytosol extraction kit (Applygen Technologies Inc., Beijing) was used to isolate the nuclear and cytosol fractions. We examined the expression levels of Nrf2 in the cytoplasm and nucleus and HO-1 in the cytoplasm. Each sample contained 50 *μ*g protein. The primary antibodies were anti-HO-1 polyclonal antibody (Santa Cruz, USA) and anti-Nrf2 polyclonal antibody (Abcam Inc., Cambridge, MA). Each protein blot was normalized to histone H3 or *β*-actin to obtain the final result expressed as intensity ratio (% sham-operated control).

### 2.8. DNA-Binding Activity Assay of Nrf2

A commercially available Active Motif's (Carlsbad, CA, USA) ELISA-based TransAM® Nrf2 kit was used to analyze Nrf2 DNA-binding activity [8]. 10 *μ*g nuclear protein was assayed per the manufacturer's protocol.

### 2.9. Determination of Protein Oxidation and Lipid Peroxidation

A commercial OxyBlot kit (#S7150; Millipore) was employed to determine the extent of protein oxidation [8]. The protein carbonyl content was used as an index for oxidative protein damage [33]. The cytoplasmic supernatant fluids of tissue samples were reacted with 2,4-dinitrophenylhydrazone (DNP) to get the DNP derivatized carbonyl groups. Then western blotting was used to test the DNP-derivatized protein with an anti-DNP antibody.

We analyzed total malondialdehyde (MDA) levels as the indicators of lipid peroxidation with the MDA detection kit (Nanjing Jiancheng, China) [8]. The absorption of the thiobarbituric acid (TBA) reactive substances which were produced by the reaction of TBA with MDA was measured at 532 nm to determine the extent of MDA. Results were presented as nmol/mg protein of wet tissue.

### 2.10. Statistical Analysis

Neurological deficits were analyzed using the Kruskal-Wallis test followed by Dunn's test, and results are expressed as median ± interquartile range. One-way analysis of variance (ANOVA) with subsequent Duncan's test as post hoc analysis was used for statistical analysis of all the other data, and results are reported as mean ± standard error (SE). A *P* value < 0.05 was considered to indicate statistical significance.

## 3. Results

### 3.1. UA Decreases Cerebral Infarct Size and Improves Neurological Functional Outcome

**Figure 1:**
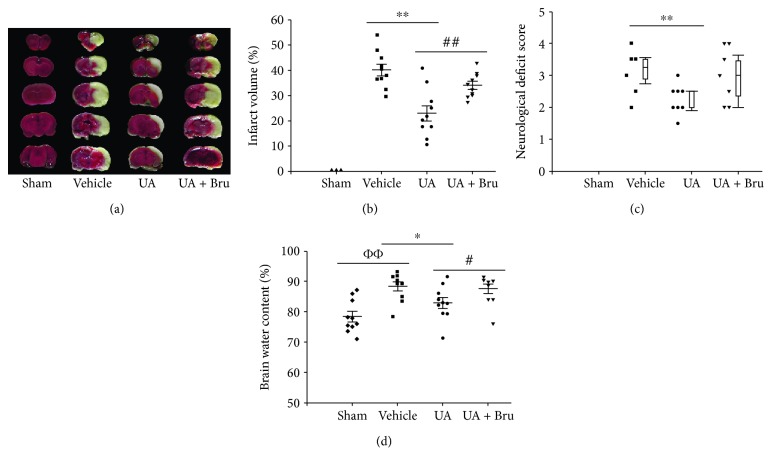
Effect of UA on the infarct volume, neurological deficit, and brain water content in rats. (a) Representative TTC-stained coronal sections. (b) Quantification of infarct volume; data are means ± SE (*n* = 10). (c) Neurological deficit scores; data are medians ± interquartile range (*n* = 10). Box plots show the interquartile range (25% to 75%) as the box, median as the horizontal line in the box, and the 95% range as the whiskers. (d) Brain water content; data are means ± SE (*n* = 12). ФФ*P* < 0.01 vs. the sham group; ^∗^*P* < 0.05, ^∗∗^*P* < 0.01 vs. the vehicle-treated I/R group; ^#^*P* < 0.05, ^##^*P* < 0.01 vs. the UA-treated I/R group.

The infarct size induced by MCAO was determined using TTC staining (Figure 1(a)). No infarct tissue was detected in the sham group, while in the vehicle group, large volumes of infarct tissue were developed. Sections from rats treated with UA had significantly decreased infarct volumes (22.93% ± 3.04%) compared to the vehicle-treated I/R group (40.18% ± 2.30%, *P* < 0.01). In order to evaluate the role of Nrf2 pathway in UA-induced neuroprotection, the effect of brusatol on cerebral infarct volume was also detected. As observed in the UA + Bru group, a larger cerebral infarct volume was presented compared with the UA-treated I/R group (Figures 1(a) and 1(b)). Neurological deficit scores are presented in Figure 1(c). For vehicle-treated I/R animals, the median score was 3.25 (interquartile range (IQR): 2.88 to 3.50), indicative of severe neurological injury. When compared to the vehicle-treated I/R group, treatment with 16 mg/kg UA (median score: 2, IQR: 2 to 2.50) decreased the neurological deficit score significantly. The brain water content decreased significantly in UA-treated rats compared with model rats (83.0 + 1.8% vs. 88.5 + 1.5%, *P* < 0.05), whereas brusatol attenuated the decrease (87.7 + 1.6%, *P* < 0.05, Figure 1(d)).

### 3.2. UA Attenuated Neuronal Injury

**Figure 2:**
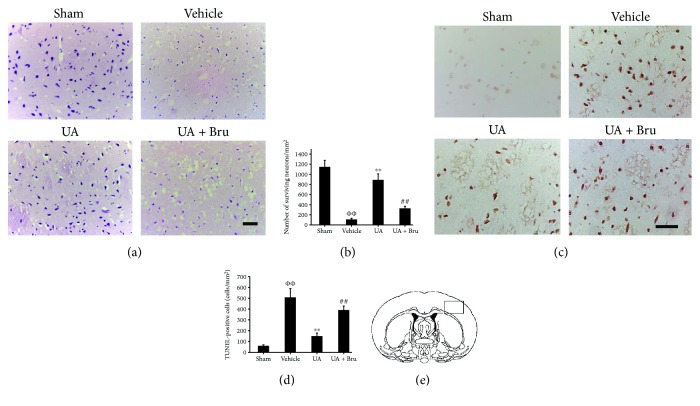
Effect of UA on neuronal injury in cerebral cortices in rats. (a) Representative photomicrographs of HE staining. Scale bar in all panels = 50 *μ*m. (b) Quantification of surviving neuronal cells. (c) Representative photomicrographs of TUNEL staining. Scale bar in all panels = 50 *μ*m. (d) Quantification of apoptotic cells. (e) Schematic diagram of coronal brain section. Black rectangle in the ischemic ipsilateral parietal cortex of penumbra represents the region selected for histology. Data are means ± SE (*n* = 3). ФФ*P* < 0.01 vs. the sham group; ^∗∗^*P* < 0.01 vs. the vehicle-treated I/R group; ^##^*P* < 0.01 vs. the UA-treated I/R group.

HE staining was an effective way to evaluate neuronal cell death. In the sham group, no significant neuronal damage or neuronal loss was observed. A significant loss of neuronal cell was observed in model rats compared with sham rats (1150 ± 130 cells/mm^2^ vs. 110 ± 23 cells/mm^2^, *P* < 0.01). More intact cells in UA-treated rats were observed in comparison with model rats owing to the neuroprotection of UA (890 ± 120 cells/mm^2^, *P* < 0.01, Figures 2(a) and 2(b)). Pretreatment with brusatol markedly abolished the protection of UA.

Apoptotic cells were identified by TUNEL staining. The apoptotic cell had round and shrunken shapes and the nuclei were darkly stained. Only few TUNEL-positive cells were observed in sham rats. TUNEL-positive cells increased significantly in model rats compared with sham rats (510 ± 80 cells/mm^2^ vs. 60 ± 10 cells/mm^2^, *P* < 0.01). TUNEL-positive cells were markedly reduced by UA administration (150 ± 29 cells/mm^2^, *P* < 0.01). However, a marked increase of TUNEL-positive cells was observed in the UA + Bru group (390 ± 38 cells/mm^2^, *P* < 0.01), suggesting brusatol pretreatment attenuated neuroprotection of UA (Figures 2(c) and 2(d)).

### 3.3. UA Alleviated Oxidative Injury

**Figure 3:**
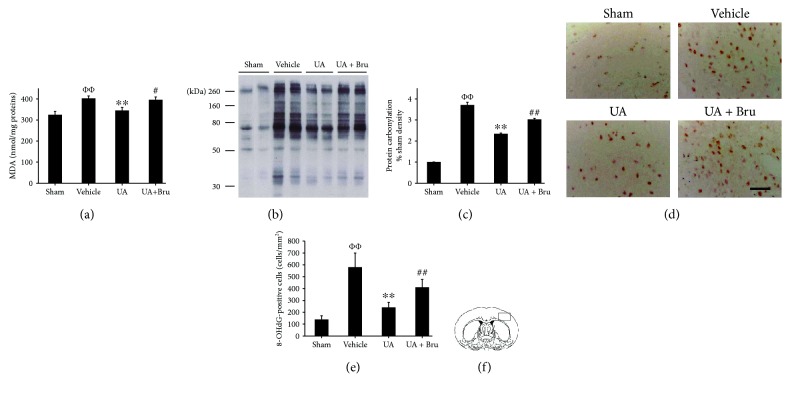
Effect of UA on oxidative injury in cerebral cortices in rats. (a) Quantification of MDA; data are means ± SE (*n* = 12). (b, c) Representative blot of protein oxidation. Quantification of carbonyl group bands; data are means ± SE (*n* = 4). (d) Representative photomicrographs of 8-OHdG immunostaining. Scale bar in all panels = 50 *μ*m. (e) Quantification of 8-OHdG-positive cells; data are means ± SE (*n* = 3). (f) Schematic diagram of coronal brain section. The black rectangle in the ischemic ipsilateral parietal cortex of the penumbra represents the region selected for histology. ФФ*P* < 0.01 vs. the sham group; ^∗∗^*P* < 0.01 vs. the vehicle-treated I/R group; ^#^*P* < 0.05, ^##^*P* < 0.01 vs. the UA-treated I/R group.

We examined the role of UA in postischemic lipid peroxidation injury by determining the concentration of MDA in ischemic side cerebral cortex. The extent of MDA increased 1.2-fold compared with the sham group. With UA administration, the MDA levels significantly decreased compared with the model group. However, the decrease was ablated by brusatol pretreatment (Figure 3(a)).

The extent of protein oxidation was measured by the levels of protein carbonylation. The protein carbonylation levels of the model rats significantly increased compared to those of the sham rats, while UA treatment decreased them compared with the model rats (*p* < 0.01, Figures 3(b) and 3(c)). The improving effect of UA on protein carbonylation levels was diminished in the ischemic cortex via pretreatment with brusatol.

Few 8-OHdG-positive cells were observed in sham rats, while a significant amount of 8-OHdG-positive cells was presented in the vehicle group (580 ± 120 cells/mm^2^ vs. 240 ± 45 cells/mm^2^, *P* < 0.01, Figures 3(d) and 3(e)). UA treatment significantly decreased the amount of 8-OHdG-positive cells. However, preadministration of brusatol significantly increased the levels of 8-OHdG immunoactivity compared with that in the UA-treated group (410 ± 67 cells/mm^2^ vs. 240 ± 45 cells/mm^2^, *p* < 0.01, Figures 3(d) and 3(e)).

### 3.4. UA Activated the Nrf2 Pathway

**Figure 4:**
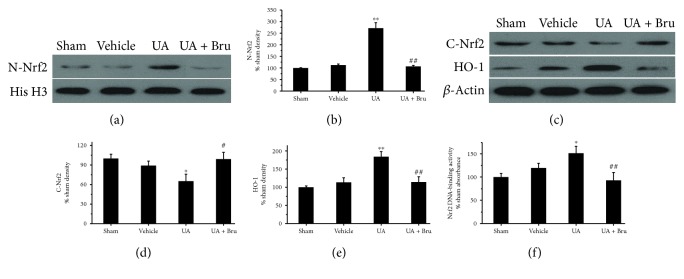
Effect of UA on Nrf2 protein distribution, HO-1 protein expression, and Nrf2 DNA-binding activity in cerebral cortices in rats. (a, b) Representative western blot of nuclear Nrf2. Quantification of nuclear Nrf2 normalized with histone H3. (c, d) Representative western blot of cytosolic Nrf2 and HO-1. Quantification of cytosolic Nrf2 and HO-1 normalized with *β*-actin. (e) Nuclear Nrf2 DNA-binding activity. Data are means ± SE (*n* = 4). ^∗^*P* < 0.05, ^∗∗^*P* < 0.01 vs. the vehicle-treated I/R group; ^#^*P* < 0.05, ^##^*P* < 0.01 vs. the UA-treated I/R group.

To find out whether the Nrf2 pathway was involved in the neuroprotection of UA, we detected the expression levels of proteins related to Nrf2 pathway carefully in the cerebral cortices of variously treated animals in the presence of brusatol. UA treatment significantly increased Nrf2 levels in the nucleus, with a corresponding decrease in the cytoplasm, compared with the vehicle group. Further studies showed that cotreatment with UA and brusatol significantly attenuated the nuclear translocation of Nrf2 (*P* < 0.05 or *P* < 0.01, Figure 4).

We next examined whether the expression of HO-1 was induced by UA. HO-1 levels were obviously increased by UA treatment, as compared to the vehicle group, an effect that was reversed by pretreatment with brusatol (*P* < 0.05 or *P* < 0.01, Figures 4(c) and 4(e)).

Furthermore, DNA-binding activity of Nrf2 was also analyzed. UA treatment enhanced Nrf2 transcriptional activity; however, brusatol blocked the increased Nrf2 DNA-binding activity of UA significantly (*P* < 0.01, Figure 4(f)).

### 3.5. UA Upregulated Expression of BDNF and NGF in Ischemic Rat Brain

**Figure 5:**
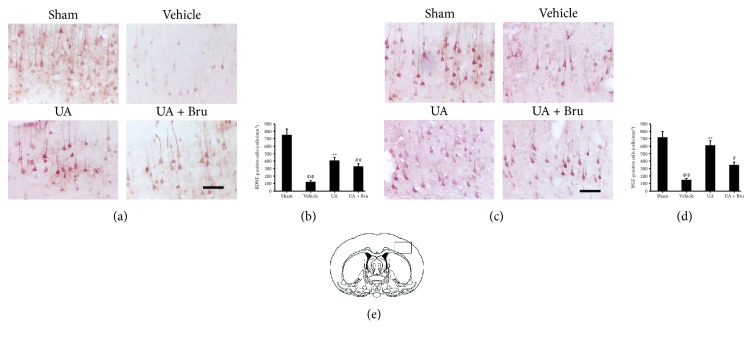
Effect of UA on the expression of BDNF and NGF in cerebral cortices in rats. (a) Representative photomicrographs of BDNF immunostaining. Scale bar in all panels = 50 *μ*m. (b) Quantification of BDNF positive cells. (c) Representative photomicrographs of NGF immunostaining. Scale bar in all panels = 50 *μ*m. (d) Quantification of NGF positive cells. (e) Schematic diagram of coronal brain section. The black rectangle in the ischemic ipsilateral parietal cortex of the penumbra represents the region selected for histology. Data are means ± SE (*n* = 3). ФФ*P* < 0.01 vs. the sham group; ^∗∗^*P* < 0.01 vs. the vehicle-treated I/R group; ^#^*P* < 0.05 vs. the UA-treated I/R group.

We examined whether UA had any effect on BDNF and NGF protein expressions using immunohistochemistry. The numbers of BDNF-immunopositive cells and NGF-immunopositive cells significantly decreased in vehicle animals; however, UA treatment successfully restored BDNF and NGF expressions, as indicated by an elevated number of BDNF-immunopositive cells and NGF-immunopositive cells (*P* < 0.01). In addition, cotreatment with UA and brusatol decreased BDNF and NGF expressions comparable to the UA-treated group (*P* < 0.05, Figure 5).

## 4. Discussion

In this study, where SD rats were exposed to FCI/R, we found that UA treatment (16 mg/kg, i.v.) decreased cerebral infarct volume, neurological deficit core, and degree of brain edema, consistent with the previous studies [23–25]. The neuroprotective properties of UA are related closely to the reduction of oxidative stress known to occur during cerebral I/R. The most novel finding of our study is that UA promoted nuclear translocation of Nrf2 and increased the expression of HO-1, as well as neurotrophic factors such as BDNF and NGF. Furthermore, the neuroprotective effects induced by UA were prevented by treatment with brusatol, an inhibitor of Nrf2. These results demonstrated that UA protected the brain from I/R injury potently and Nrf2 pathway activation as well as the upregulation of BDNF and NGF expression levels might be involved in this brain protection.

The cellular injury and downstream pathway activation caused by oxidative stress may exacerbate the postischemic brain damage [2]. MDA, protein carbonyls, and 8-OHdG, which are oxidized products of lipid, protein, and DNA, respectively, are the major oxidative stress biomarkers. We observed a significant amount of MDA, protein carbonyls, and 8-OHdG in I/R rats. UA is a powerful natural antioxidant and radical scavenger that has cytoprotective effects against oxidative stress by scavenging ROS and suppressing lipid peroxidation in cultured hippocampal neurons after exposure to glutamate or cyanide [25]. In addition, UA protects the brain via reducing oxidative/nitrative stress following I/R in rats [24]. In the current study, the concentrations of MDA, protein carbonyls, and 8-OHDG were markedly decreased in rats with administration of UA. However, these changes could be largely blocked by the function of brusatol. Our results also showed that neuronal cells largely decreased with damaged morphology and the apoptotic cell death increased in the TUNEL assay in model rats. UA administration was able to improve histology changes and also attenuate TUNEL-positive cells. All these data support the view that UA treatment alleviates brain damage through reducing lipid, protein, and DNA peroxidation in cortical cells, subsequently reducing brain infarction.

Little is known about the mechanisms on how UA treatment protects neurons against oxidative stress. Evidences demonstrated that Nrf2 is a master regulator of the antioxidative defense responses [34]. Under basal conditions, Nrf2 largely localizes in the cytoplasm in an inactive form to maintain the expression of Nrf2-regulated genes in a low basal level. Under stressful conditions, Nrf2 translocates from the cytoplasm to the nucleus to bind ARE, which results in activation of transcription of multiple antioxidant and detoxifying genes [35, 36]. Our group and others have evidenced the Nrf2 pathway as therapeutic target of ischemic stroke [8, 37, 38]. Therefore, this study further detected whether UA could activate the Nrf2 pathway. UA stimulation elevated Nrf2 protein expression in the nucleus and the binding activity of Nrf2 to AREs, with a corresponding decrease in the cytoplasm, suggesting that UA treatment induced nuclear translocation of Nrf2. The structure-activity relationship may account for one of the mechanisms that urate activates Nrf2. Structurally, UA displays ketoenol tautomeric forms and can react with cysteine residues in Kelch-like ECH-associated protein 1 (Keap1), which principally regulates the protein stability and transcriptional activity of Nrf2, through adduct formation and redox modifications as an electrophilic compound, triggering Nrf2 nuclear translocation. Simultaneously, UA promoted HO-1 expression. HO-1, which is regulated by Nrf2, has been recently considered to be important against ischemic brain injury [39]. It directly affects the antioxidative balance in the body, and it has antiapoptotic activities as well [40]. Importantly, the ability to promote Nrf2/HO-1 protein expression and Nrf2 nuclear translocation of UA was significantly blocked after preadministration of brusatol, suggesting that UA may regulate ARE-related gene expression through promoting Nrf2 activation to play antioxidative effects after FCI/R. In addition, brusatol treatment suppressed the antioxidative stress activities of UA and aggravated neuronal injury after FCI/R. These data strongly demonstrated that UA activated Nrf2 signaling pathway to perform its antioxidative and antiapoptotic activities as well as neuroprotective effects. However, while we have evidenced that the protein expression of HO-1 increased via UA treatment in this study, it is noteworthy that it does not implicate that HO-1 is the only factor involved in the protective effects of UA. An in vitro study proved that UA treatment increased mRNA levels of the modifier subunit of the *γ*-glutamyl cysteine ligase (GCL) and NAD(P)H:quinone oxidoreductase 1 (NQO1) and protein levels of GCLM in astrocyte cultures, whose inductions are governed by Nrf2, suggesting other antioxidants induced by Nrf2, may also contribute to the protective effects of UA [28]. Thus, the neuroprotective actions of UA may also be due to a wide spectrum of other Nrf2-regulated antioxidant genes potentially.

In addition, our study demonstrated that UA significantly increases the endogenous BDNF and NGF expressions. Several studies indicate that increased levels of neurotrophins have a protective role in experimental stroke studies [10]. The family of neurotrophins includes BDNF and NGF. Both animal and clinical studies have shown that BDNF has an effect to improve neurological recovery, prevent neuronal death, and support the survival of many types of neurons after cerebral ischemia [12, 13, 41]. NGF is also critical for neuronal survival, proliferation, and differentiation, and it has been reported that NGF is essential to improve neurological function after cerebral ischemia [12, 42]. By binding to tropomyosin receptor kinase (Trk) receptors, BDNF and NGF activate the downstream signaling pathways such as phosphatidylinositol 3-kinase (PI3K)/protein kinase B (Akt) pathways and the mitogen-activated protein kinase (MAPK)/extracellular signal-regulated kinase (ERK) [43, 44]. Several lines of evidence reported that Nrf2 activation can be regulated via PI3K/Akt and MAPK/ERK pathways and thereby affects redox homeostasis [17, 19, 45–47]. Some studies suggested that BDNF can active antioxidant mechanisms constantly by inducing Nrf2 nuclear translocation [18]. Neurotrophic signaling pathway may be an upstream mechanism of the Nrf2 activation. Other studies have demonstrated that Nrf2 played a physiological role in the neuroprotective response to regulate BDNF and NGF expressions in a Nrf2-dependent manner which may confer neuroprotection by attenuating oxidative stress [48–50]. It is still difficult to propose the precise mechanisms that control Nrf2 translocation and how the Nrf2 antioxidant system talks to the neurotrophic signaling pathway. In the present study, the upregulation of BDNF and NGF expressions by UA was partially depressed by brusatol, suggesting that UA stimulates BDNF and NGF expressions through a Nrf2-mediated pathway. Although the precise mechanism of Nrf2-mediated induction of the BDNF and NGF expressions is still unclear, these findings implicate that augmenting the levels of neurotrophins via pharmacological strategies may be one of the factors contributing to the beneficial effects of UA, which may offer a powerful therapeutic target of oxidative damage. However, the exact events in the upregulation of neurotrophin expression by UA in the I/R rat brain need further exploration.

Our study has several limitations. First, it has been demonstrated that hyperemia at reperfusion can be a sign of worse I/R injury [51, 52]. Prior study has shown that the neuroprotective effect of UA is more marked in the rats that develop a hyperperfusion state after reperfusion [26]. However, the state of brain blood perfusion at early reperfusion was not detected in the current study. Second, sample sizes as 3-4 per group for immunochemistry and western blot detection methods are fairly small. The relatively small number of the samples often causes low statistical power, which is one of the factors related to low reproducibility of results from preclinical animal research. So sample sizes should be enlarged in the future investigation to validate our findings. And last, in the current study, 24 hours after transient FCI was chosen as the endpoint based on previous literature data as many studies reported neuroprotective effects of UA at this time point [23, 26]. The present study strengthens the concept that UA exhibits short-term antioxidant and neuroprotective effects. Further studies are essential to extend observation periods after transient FCI to detect the longer therapeutic effect of UA.

## 5. Conclusions

For the first time, this study demonstrates that UA confers neuroprotection against FCI/R-induced oxidative stress via Nrf2 pathway activation as well as BDNF and NGF expression upregulation. The protective effect of UA was blocked by Nrf2 inhibitor brusatol. Nonetheless, further study deserves to be done in the future to investigate whether other pathways or mechanisms participated in the neuroprotective effects of UA.

## Data Availability

The data used to support the findings of this study are included within the article.
